# Broad and Inconsistent Muscle Food Classification Is Problematic for Dietary Guidance in the U.S.

**DOI:** 10.3390/nu9091027

**Published:** 2017-09-16

**Authors:** Cody L. Gifford, Lauren E. O’Connor, Wayne W. Campbell, Dale R. Woerner, Keith E. Belk

**Affiliations:** 1 Department of Animal Science, Colorado State University, Fort Collins, CO 80523, USA; cody.gifford@rams.colostate.edu (C.L.G.); dale.woerner@colostate.edu (D.R.W.); 2 Department of Nutrition Science, Purdue University, West Lafayette, IN 47907, USA; leoconno@purdue.edu (L.E.O.); campbellw@purdue.edu (W.W.C.)

**Keywords:** muscle foods, assessment methods, nutrient content, dietary recommendations, classification, specification

## Abstract

Dietary recommendations regarding consumption of muscle foods, such as red meat, processed meat, poultry or fish, largely rely on current dietary intake assessment methods. This narrative review summarizes how U.S. intake values for various types of muscle foods are grouped and estimated via methods that include: (1) food frequency questionnaires; (2) food disappearance data from the U.S. Department of Agriculture Economic Research Service; and (3) dietary recall information from the National Health and Nutrition Examination Survey data. These reported methods inconsistently classify muscle foods into groups, such as those previously listed, which creates discrepancies in estimated intakes. Researchers who classify muscle foods into these groups do not consistently considered nutrient content, in turn leading to implications of scientific conclusions and dietary recommendations. Consequentially, these factors demonstrate a need for a more universal muscle food classification system. Further specification to this system would improve accuracy and precision in which researchers can classify muscle foods in nutrition research. Future multidisciplinary collaboration is needed to develop a new classification system via systematic review protocol of current literature.

## 1. Introduction

Muscle foods (i.e., skeletal muscle and the associated tissues) are broadly categorized into red meat, poultry, fish and processed meat. Organizations that release public dietary guidelines, such as the World Cancer Research Fund [1] and the U.S. Department of Agriculture (USDA) via the Dietary Guidelines for Americans [2], consistently define these categories as originating from a list of animal species (e.g., beef, chicken, tuna, etc.) in an attempt to simplify the products in an easy-to-understand format for consumers. In all cases, red meat is referred to as mammalian meat, most commonly noted as beef, pork, and lamb. While some researchers consider white meat to be inclusive of poultry (muscles from birds) and fish (muscles from aquatic animals) [3,4,5], the term “white meat” rarely appears in dietary guidelines. Processed meat is defined as red meat and poultry, but not often fish, which are cooked further or contain preservatives. Some muscle foods meet qualifications to fall into more than one category, for example deli turkey meat is both poultry and processed meat. This introduces the need for more comprehensive subcategories of muscle foods in dietary guidelines.

There are more detailed muscle food group classification systems available. From a regulatory perspective, the USDA Food Safety and Inspection Service provides technical definitions for muscle foods due to the need to provide standards of identity [6]. Meat scientists argue that the simplicity of regulatory definitions do not accurately reflect differences in physical characteristics, degree/type of processing, and most notably, nutrient content. This led to the American Meat Science Association’s (AMSA) development of a Meat Lexicon, which provides detailed classifications of meat, poultry, and fish products [7]. This lexicon separates muscle foods into “minimal processing” and “further processing” with four subcategories within those main groups. While comprehensive, collecting dietary intake data in nutrition research studies with this level of complexity is not obtainable with the current dietary intake assessment methods.

Researchers commonly assess even broader muscle food categories than those presented previously, such as “red and processed meat”, with little to no justification or clarification. This is problematic because the nutrient content can substantially vary in those muscle food categories. Inconsistent muscle food categorization creates discrepancies in the U.S. about the quantity and types consumed. Without accurate knowledge of muscle food intakes, it is difficult to study the relationships between muscle food intake and disease risk to create public dietary guidelines. Biomedical researchers and public health policy-makers advocate for more detailed and consistent definitions and categorizations in nutrition research in order to provide public dietary guidance that carefully considers the nutritional differences of muscle food subcategories [8].

To accomplish our objectives, we constructed a narrative review as a means to demonstrate the current methods utilized for estimating dietary intake of muscle foods in the U.S. In this narrative review, we discuss how muscle food types are grouped and intake values estimated in the U.S. via: (1) food frequency questionnaires; (2) food disappearance data; and (3) dietary recalls. We highlight the importance of considering nutrient content when grouping muscle foods during intake assessment by discussing the groupings used in the Harvard Food Frequency Questionnaire (FFQ) as an example. Lastly, we discuss implications of inconsistent meat groupings on scientific conclusions and dietary guidelines and emphasize the need for a systematic review of current literature to design a new system to better classify muscle foods.

## 2. How Muscle Food Types Are Grouped and Intake Values Estimated in the U.S.

Detailed dietary intake assessments are critical to nutrition research and dietary guidance but are largely lacking. Current dietary intake assessment methods include food disappearance data from the USDA Economic Research Service (USDA-ERS) [9], FFQs (such as the Harvard T.H. Chan School of Public Health and the Brigham and Women’s semi-quantitative food frequency questionnaire (Harvard FFQ) [10] and the National Cancer Institute Diet History Questionnaire II (NCI FFQ) [11]), and dietary recalls from the National Health and Nutrition Examination Survey Food Patterns Equivalents Database (NHANES, FPED) [12] (see Table 1 for more information about these methods). Food disappearance data overestimate intakes of agricultural commodities due to unmeasurable losses such as food spoilage and plate waste [13], which account for approximately 31% of the total food supply [14,15]. On the contrary, self-reported dietary intakes are often underreported by as high as 20% in dietary recalls and 38% in FFQs [16]. Food frequency questionnaires ask respondents about their habitual consumption of broad food categories to yield approximate intakes while dietary recalls can obtain a more sophisticated level of detail via comprehensive interviewing. However, NHANES-FPED dietary recall data are coded into broad food categories for public use. Because these datasets are broadly grouped, detailed assessment of muscle food subcategories is limited.

The USDA-ERS and NHANES, FPED datasets [9,12], which are both federally managed, classify muscle food products differently into broad categories. These differences are highlighted in Table 2. The NHANES, FPED category of “meat” refers only to red meat, inclusive of beef, veal, pork, lamb, and game. The “total red meat” category in the USDA-ERS dataset [12] excludes meat from game animals. The NHANES, FPED dataset includes game birds under the “poultry” category, but meat from all game animals is not accounted for in the USDA-ERS data. Additionally, the two datasets refer to the “total” categories differently. The NHANES, FPED categorizes “total meat” as “meat”, “cured meat”, “organ meat”, “poultry”, “seafood from low omega-3 sources” and “seafood from high omega-3 sources”. The USDA-ERS includes the “total red meat” subcategory as part of the “total meat, fish, eggs and nuts” category along with “total poultry”, “total fresh and frozen, canned, and cured fish and shellfish”, “eggs”, “peanuts”, “total tree nuts” and “coconut” (Table 2). However, one cannot access the data for the “total meat” subcategory independent of the others mentioned. Differences in these categories limit the comparison of estimated muscle food intakes across these two methods.

**Figure 1:**
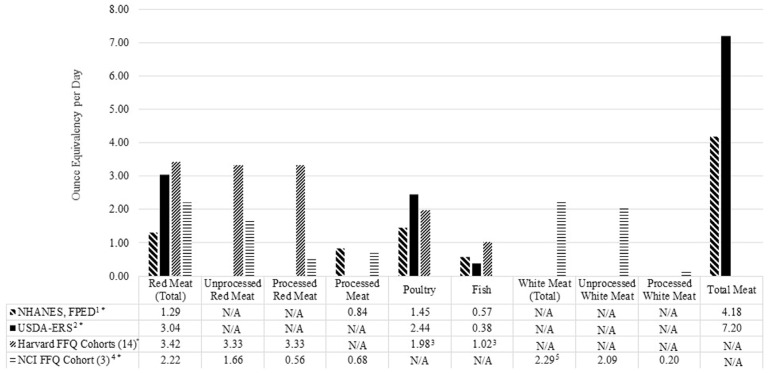
Comparison of muscle food groupings and estimated intakes (ounces *) from food frequency questionnaires (FFQ), food disappearance data, and dietary recalls. ^1^ Adapted from United States Department of Agriculture, Agricultural Research Service, National Health and Nutrition Examination Survey, What We Eat in America, Food Pattern Equivalency Database 2013–2014 dataset available at [12] ^2^ Adapted from United States Department of Agriculture, Economic Research Service, Food Availability (Per Capita) Data System, Loss-Adjusted Food Availability, Meat, poultry, fish, eggs, and nuts, and fish. USDA-ERS per capita availability reflects supply at the consumer level adjusted for loss from primary, retail and consumer sources of loss or waste. More information about adjusting for loss can be found at [9]. ^3^ Weighted means were calculated for the median quartile of intake based on a three-ounce serving. ^4^ Baseline pooled median quartile intake was reported as g/1000 kcal from the National Institute of Health American Association of Retired Persons (NIH-AARP) cohort that used the National Cancer Institute Diet History Questionnaire II (NCI FFQ); intake levels presented in this figure are calculated ounce equivalents converted from g/2000 kcal. ^5^ White meat in the NIH-AARP cohort is inclusive of poultry and fish. N/A = Data not available. * 1 ounce ≈ 28 g.

Questions posed in FFQs address broad muscle food groups which limit researchers’ ability to separate these groups into more detailed subcategories. To further understand these inconsistencies, estimated intakes among all methods previously described are presented in Figure 1. Previous studies that utilized FFQs reported “unprocessed red meat” and “processed red meat” separate from “total red meat” in an attempt to gain a more sophisticated level of detail [3,17]. However, it is unclear how researchers calculate “unprocessed red meat”, specifically intake using the Harvard FFQ, because the questions do not address unprocessed red meat independently. Rather, the questions address broad categories of “pork” or “beef or lamb” as a main dish which can include both processed and unprocessed cuts of red meat. The NCI FFQ [11] elicits more detail from respondents about processed red meat and poultry products than the Harvard FFQ [10]. However, researchers are still at liberty to group these data as they see fit for analysis and presentation. For example, the NCI FFQ reference [3] in Figure 1 reports liver as an “unprocessed red meat” while the Harvard FFQ does not include organ meat in this category [17]. Additionally, the researchers using the NCI FFQ group poultry and fish as “white meat” even though the term “white meat” is not commonly used by other resources. Further, foods within these groups can have wide variations in nutrient content which will be addressed in detail later in this review. 

The muscle foods included in these broad inconsistent categories need to be carefully defined by researchers when estimating intakes. For example, the inclusion of non-muscle foods, mentioned previously in the USDA-ERS “total” category, are most likely driving the estimated “total” intake to be twice as high as the NHANES estimated “total” intake shown in Figure 1. The NHANES, FPED data separate “cured meat” from the “meat”, “poultry”, and “seafood” categories while the USDA-ERS does not. This is a potential driver for the USDA-ERS red meat and poultry intake estimates to be about twice as high as NHANES, FPED estimates even after adjusting for losses. Even intake estimates using the same method can lack consistency. For example, the categorization difference in FFQ limits the ability to compare poultry and fish intakes independently because the resource using the NCI FFQ listed in Figure 1 groups them into a “white meat” category. These nuances in inconsistent muscle group categorizations, along with methodological limitations, lead to different intake estimates.

**Figure 2:**
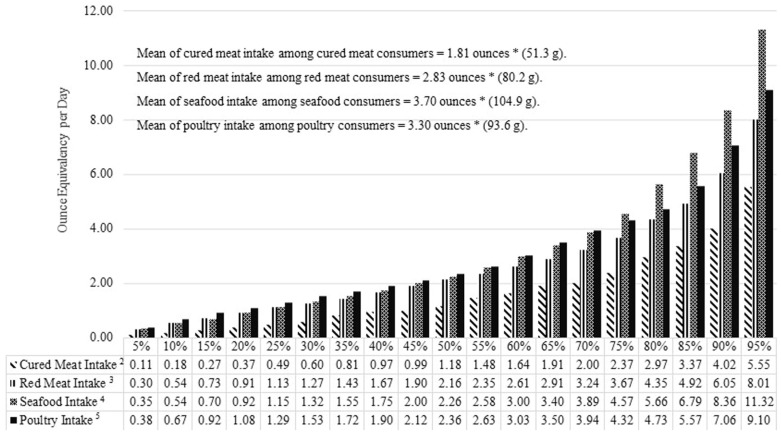
Intake distribution of red meat, cured meat, seafood and poultry, among U.S. respondents from the National Health and Nutrition Examination Survey Food Pattern Equivalency Database (NHANES, FPED) 2013–2014 Datasets ^1^. ^1^ Adapted from United States Department of Agriculture, Agricultural Research Service, NHANES, WWEIA, FPED 2013–2014 available at [12]. ^2^ Intake of red meat from red meat consumers in this dataset. ^3^ Intake of processed meat from processed meat consumers in this dataset. ^4^ Intake of seafood from seafood consumers in this dataset. ^5^ Intake of poultry from poultry consumers in this dataset. * 1 ounce ≈ 28 g.

The upper and lower extreme percentiles can also skew estimated mean intakes. The reference using the Harvard FFQ [17] estimates “total red meat”, “unprocessed red meat” and “processed red meat” intakes 2–3-times higher than the reference using the NCI FFQ [3] and NHANES, FPED datasets. This is most likely because the Harvard FFQ data are collected from omnivores only [17] while the NHANES, FPED [12] and the NCI FFQ data [3] include vegetarians. Because of this large discrepancy in intakes, we also estimated these categories in NHANES-FPED dataset excluding vegetarians, as shown in Figure 2. Among all participants in the 2013–2014 NHANES, FPED dataset, only 38%, 40%, 39%, and 7% of respondents reported consuming any “meat”, “cured meat”, “poultry” and “seafood”, respectively. About 70% of red meat consumers, 80% of cured meat consumers, 60% of fish consumers, and 60% of poultry consumers reported intakes of 3 ounces (85 g) or less of each respective muscle food. Daily intake levels of the majority of muscle food eaters are modest, but there are large jumps at the 90th percentile, especially in red meat and fish consumption. For example, estimated seafood intake at the 95th percentile is almost double compared to seafood intake from 85th percentile of this subpopulation. When assessing muscle food group intakes, accounting for the upper and lower extreme percentiles will lead to a better representation of average muscle food consumers.

Estimating muscle food intakes proves to be a challenging task, complicated by methodological limitations, inconsistent muscle food groupings, and extreme upper and lower percentiles. However, food disappearance data, dietary recalls, and FFQs are the best dietary data collection methods currently available. Until new more accurate methods or technologies become available, these limitations will continue to compromise estimated muscle food intakes.

## 3. The Importance of Considering Nutrient Content When Grouping Muscle Foods for Dietary Intake Assessment

**Figure 3:**
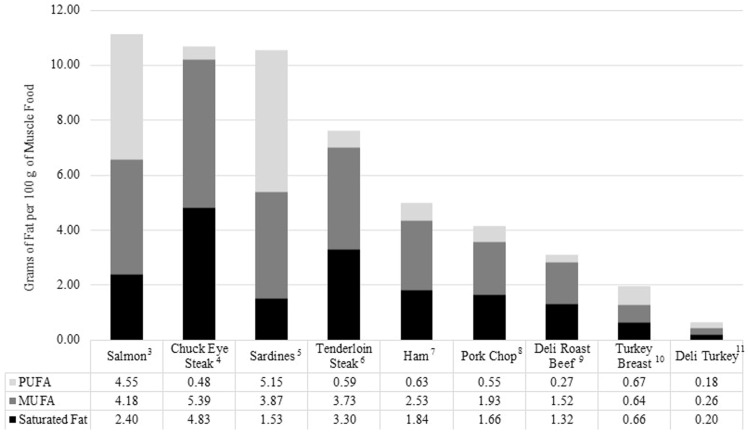
Fatty acid profiles (g of fat per 100 g ^1^) of selected cooked unprocessed and processed muscle foods ^2^. ^1^ 9 CFR 317.362 USDA: Lean classifications per 100 g include and are defined as (1) Lean: <10 g total fat, <5 g saturated fat, <95 mg cholesterol; available at [20]. ^2^ Adapted from United States Department of Agriculture, Agricultural Research Service, Food Composition Database available at [18]. ^3^ USDA-ARS Food Composition Database number 15237: Fish, salmon, Atlantic, farmed, cooked, dry heat. ^4^ USDA-ARS Food Composition Database number 23077: Beef, chuck eye steak, boneless, separable lean only, trimmed to 0’ fat, all grades, cooked, grilled. ^5^ USDA-ARS Food Composition Database number 15237: Fish, sardine, Atlantic, canned in oil, drained solids with bone. ^6^ USDA-ARS Food Composition Database number 13442: Beef, loin, tenderloin steak, boneless, separable lean only, trimmed to 0’ fat, all grades, cooked, grilled. ^7^ USDA-ARS Food Composition Database number 10067, Pork, fresh, loin, top loin (chops), boneless, separable lean only, cooked, braised. ^8^ USDA-ARS Food Composition Database number 10153: Pork, cured, ham, whole, separable lean only, roasted. ^9^ USDA-ARS Food Composition Database number 07043: Roast beef, deli style, prepackaged, sliced. ^10^ USDA-ARS Food Composition Database number 05711, Turkey, retail parts, breast, meat only, cooked, roasted. ^11^ USDA-ARS Food Composition Database number 07046: Turkey breast, low salt, prepackaged or deli, luncheon meat.

As mentioned previously, FFQs estimate intakes of broad muscle food groups but the muscle foods included in these groups can span a wide spectrum of nutrient contents. In this section, we present questions from the Harvard FFQ to demonstrate this. We discuss specific questions that are posed to respondents about muscle food groups (see Table 3) and provide examples of foods within these groups, mainly regarding red and processed meat, which can vary substantially in nutrient content, as shown in Table 4 and Figure 3.

The Harvard FFQ mainly focuses on separating muscle foods by species which is appropriate due to varying differences in total and saturated fat content among numerous muscle foods. For example, Table 4 shows that a beef tenderloin steak, although considered a lean meat by USDA regulatory standards for labeling claim purposes [20], contains 5 g more total fat than fresh cooked turkey breast (on a 100 g cooked basis). To further highlight this point, Figure 3 shows that turkey breast contains <1 g of saturated fat while the red meat cuts contain up to 5 g (on a 100 g cooked basis), yet these products are still considered lean. The Dietary Guidelines for Americans recommend consuming <35% and <10% of total energy from total and saturated fat, respectively [2]. It is common to recommend limiting red meat intake to meet these goals and therefore logical to address red meat consumption separate from poultry in prospective cohort studies relating muscle food intake to disease risk.

There are a variety of lean red meat options [21,22] currently available to consumers which can help meet these recommendations, but the Harvard FFQ does not address lean red meat consumption. Only two questions in the Harvard FFQ (9 and 10 in Table 3) address fat content in regard to a “lean”, “extra lean”, or “regular” hamburger. Definitions of “lean”, “extra lean”, or “regular” are not provided so the respondents are likely to provide varied responses based on personal perception. The other questions about red meat group lean and non-lean red meat together. For example, question 13 in Table 3 asks about consumption of “beef or lamb as a main dish, e.g., steak, roast”. Based on regulatory standards, beef tenderloin and deli roast beef meet federal requirements to be labeled as lean (per 100 g: <10 g total fat, <5 g saturated fat, <95 mg cholesterol), but beef chuck eye steak does not [20]. Although it is often recommended to limit red meat consumption due to higher total and saturated fat content compared to some poultry and fish, a third of the saturated fat in red meat is stearic acid which has a neutral effect on health biomarkers [23]. Additionally, red meat has a higher proportion of monounsaturated fatty acids than poultry or fish. Questions addressing intakes of lean and non-lean sources of red meat would be beneficial in FFQs. Lean red meats are nutrient dense foods which can be part of a healthy eating pattern, according to the 2015 Dietary Guidelines for Americans [2].

It is difficult to completely parse out processed vs. unprocessed muscle foods using the Harvard FFQ which is important due to variations in micronutrient content. Specifically, questions 2, 11 and 19 query about the consumption of “chicken or turkey sandwich or frozen dinner”, “pork as a main dish, e.g., ham or chops”, and “dark meat fish, e.g., tuna steak, mackerel, salmon, sardines, bluefish, swordfish”, respectively. A turkey sandwich could contain fresh turkey breast or processed turkey deli meat, ham is most commonly cured while chops are unprocessed, and sardines are preserved with salt while salmon is commonly consumed fresh. Unprocessed turkey breast and pork chops contribute 24–45% less sodium and 23–49% more niacin to the diet than deli turkey and ham, respectively. Similarly, salmon contributes 9% less sodium, 9% more niacin, and 12% less iron to the diet compared to sardines. Although these processed meats contribute adequate amounts of zinc, potassium, niacin and vitamin B_12_ while remaining low in fat, the sodium content is much greater than unprocessed meat. The high sodium content of these processed meats may overshadow the health benefits of consuming their non-processed counterparts included in these questions.

The degree and type of processing result in micronutrient variations within the same species. For example, a three-ounce portion of beef tenderloin and beef chuck eye steak contribute only 2–3% of the daily value of sodium which is 28% less than deli style roast beef (Table 4). These unprocessed beef products contribute greater amounts of zinc (27–60% of the daily value) and vitamin B_12_ (48–65% of the daily value) compared to deli style roast beef. In further comparisons to most poultry and fish products, beef chuck eye steak and beef tenderloin contribute at least 10% or greater of the daily value of iron, zinc, niacin and vitamin B_12_ (Table 4). Unprocessed red meat is a superior source of several essential micronutrients compared to processed red meat, as well as most poultry and fish products [23,24,25,26,27], further emphasizing the importance of considering micronutrient content in assessment of dietary intake. 

In some cases, it is difficult for researchers to classify muscle food products as “processed” or “unprocessed” due to the simplicity of definitions. For example, processed foods are defined as whole foods that are altered from their original state, sometimes including the addition of other ingredients [28,29]. Regardless of fat level, ground meat is altered by grinding whole muscles (e.g., beef, pork, turkey, etc.) into a comminuted final product [30], but has no non-meat ingredients. Further, the term “processed meat” is often used synonymously with “cured meat” [30] which refers to a processing step of adding nitrite as an ingredient according to several cured meat definitions [31,32]. However, new technologies allow the production of frankfurters using celery juice [31] rather than sodium nitrite. As a result, products such as ground meat or no-nitrite added frankfurters fall into a gray area and are difficult to classify as “processed” or “unprocessed” meat. 

Lean unprocessed muscle foods have nutrient compositions that can be incorporated into a healthy eating pattern if consumed in moderation. For example, three ounces (85 g) of most red meat contains ~20 g of protein and ~200 calories with sufficient micronutrient content [25,33]. In comparison, walnuts [34] contribute 860 calories and approximately 86 g of total fat to provide 20 g of protein [18]. However, over consumption of non-lean and processed muscle foods can lead to daily values above 100%, particularly for individuals above the 80th consumption percentile. For example, a nine-ounce steak contributes 25% of daily energy and ≥20 g of fat, and a nine-ounce serving of ham contribute 3384 mg or 141% of the daily value for sodium. Therefore, a more detailed assessment of muscle food intakes would help formulate more comprehensive omnivorous eating patterns with quantifiable and specific muscle food recommendations. 

It is important for researchers to be able to measure lean, non-lean, processed, and unprocessed muscle food intakes due to differences in macro- and micronutrient contents. The current methods available, specifically the Harvard FFQ described in this section, have limited capabilities of achieving this. There are relevant nutrient variations and processing methods across and within red meat, processed meat, poultry, and fish which can influence conclusions about the health effects or associations of consuming these foods. For researchers specifically interested in the disease risk associated with muscle food consumption, development of a muscle food-specific FFQ is warranted to address fat levels and degrees/types of processing. This proposed FFQ would provide descriptive definitions of “lean” and “processed” muscle foods and assess different types of processed muscle foods (including cured, smoked, different types of preservatives, etc.) so respondents do not have to rely on personal perceptions. Commercially available meat products are regularly being improved with lower fat or sodium options and different types of preservatives therefore frequent updates of this proposed FFQ is warranted. This would provide more detailed muscle food intake assessments to help formulate more specific and quantified dietary guidelines. 

## 4. Discussion of Implications of Inconsistent Muscle Food Groupings on Scientific Conclusions and Dietary Guidelines

The broad grouping of muscle foods in dietary intake assessment methods, noted in previous sections, have large-scale implications in nutrition research and dietary guidelines. Dietary guidance regarding muscle food intake is largely based on prospective cohort studies relating FFQ data to health outcomes. The broad spectrum of meat, poultry and fish products included in FFQ questions can complicate responses due to wide variation in nutrient content. It is not always clear how to separate these questions into processed, unprocessed, lean, non-lean, etc. Therefore, researchers are left to interpret the data as they see fit. 

Most commonly in nutrition research, “red and processed meat” is a single grouping when assessing disease risk related to consumption of red meat. This is especially problematic when assessing cardiovascular disease risk due to differences in sodium content of unprocessed red meat vs. processed meat, as discussed previously, and the effect of sodium on increasing blood pressure [35,36]. Although the combination of unprocessed red meat and processed meat is common in the Harvard FFQ, there is little justification to combining these foods. We calculated correlations using the 2013–2014 NHANES, FPED dataset discussed previously. Among meat consumers, red meat intake was not correlated with cured (processed) meat intake (*r*^2^ < 0.002). Mean cured meat intake was 0.7 ounce per day among poultry consumers and 0.8 ounce per day among red meat consumers. Therefore, high red meat intake does not equate to high processed meat intake suggesting that “red and processed meats” is not a suitable category.

Some muscle food products, such as sandwich meat, are inconsistently reported as processed or unprocessed meat. Here we discuss meta-analyses that assess the risk of consuming 100 g of unprocessed red meat per day on cardiovascular disease risk to demonstrate how inconsistent classifications of specific meat products can result in different conclusions. While researchers’ definitions of red and processed meat (if provided) listed in Table 5 are relatively consistent, the way in which some meat products are categorized based on these definitions still varies. For example, one study by Pan et al. (2012) classifies red meat consumed in sandwiches as “unprocessed red meat” [37]. The other analyses in Table 5 [38,39,40] classify sandwich meat as deli or luncheon meat which are processed meat according to most public health definitions [1,2,6,41]. Pan et al. (2012) concludes that consuming 100 g per day of unprocessed red meat increased cardiovascular-related mortality by 18% [37]. However, the other analyses in Table 5, which classifies sandwich meats as “processed meat”, shows no increase in cardiovascular-related mortality [40] or risk of cardiovascular disease events [38,39,42] from consuming 100 g of unprocessed red meat per day. These categorization nuances can lead to erroneous conclusions about the health implications of consuming unprocessed red meat.

Difficulties in interpreting these inconsistent meat categories throughout the scientific literature are highlighted in the 2015 Dietary Guidelines Advisory Committee (DGAC) Scientific Advisory Report. The DGAC concluded that the “meat group” had the most variability in terminology compared to all other food groups. Examples of meat categorization variability are described previously in this review (see Table 2). During the evidence review process, the DGAC attempted to adhere to the language used by individual researchers, but then provided a general label of “meat” in the final eating pattern models. “Meat” is presumably red meat because it is a subcategory of “protein foods” separate from “poultry”, “seafood”, “eggs”, “nuts/seeds”, and “processed soy” but it is not labeled as “red meat” [8].

Although this issue gained attention when the 2015 DGAC included the disclaimer about meat terminology in the Scientific Advisory Report, this issue is apparent in previous DGAC reports and DGA policy documents. As shown in Table 6, the term “meat” was categorized with various other protein sources. “Meat” is its own category only in the 7th edition of the DGA released in 2010 because there are clear separate categories for poultry and fish. In the 6th edition of the DGA released in 2005, the “meat and bean” group encompasses all plant and animal protein sources, except dairy. The 2015 DGA policy document is the first to provide a definition of “meat” in the glossary, which was: “also known as red meat” including “all forms of beef, pork, lamb, veal, goat, and non-bird game (e.g., venison, bison, elk)” [8]. However, the term “red meat” does not appear anywhere else in the document or in previous editions.

Inconsistent categorization of muscle foods, specifically red and processed meat, in nutrition research can increase bias and cause inconsistent associations between muscle food consumption and disease risk. Limitations with the current methods for estimating intake of muscle foods described in this narrative review may apply to other food categories or food groups. Further research using a systematic review protocol is needed to confirm this in other food groups such as dairy. Research is also needed in the U.S. to address the impact of the multifaceted food system on the nutritional value of meat. Researchers recognize the supply chain, storage, and processing as facets that can affect nutrition quality in other food groups [43]. Ultimately, a universal and functional muscle food classification system is warranted for nutrition researchers. This will require extensive review and collaboration, but will decrease the variability in research methods, conclusions, and public health messages.

## 5. Future Directions and Conclusions

Inconsistent muscle food grouping is a reoccurring issue in nutrition research and public dietary guidelines in the U.S. How muscle foods are classified into broad categories such as red meat, poultry, or fish differs among dietary intake assessment methods including food disappearance data, dietary recalls and FFQs. The AMSA Meat Lexicon is one resource developed to apply more standardization and specification to this complex topic [7]. However, it is unclear as to which types of studies would be able to incorporate this level of detail, especially among current dietary intake assessment methods available. 

Limitations and inconsistencies within and across these dietary intake assessment methods lead to discrepancies in estimated intakes warranting more detailed muscle food-specific intake assessment methods. Information in this narrative review emphasizes the need for an updated muscle food classification system. This task would appropriately be accomplished by convening an expert panel and using a systematic review protocol of previous literature. New methods need to consider nutrient content, leanness, degree of processing, and specific types of processing. Adding further specification to the classification systems utilized by researchers would improve the accuracy of characterizing muscle foods in research. New muscle food-specific intake methods would lessen the inconsistencies noted in nutrition research when relating muscle food intakes to disease risk. This could lead to more comprehensive and quantitative dietary guidelines regarding muscle food categories. However, further research is needed to compare and modify classification systems used in prospective cohort studies as well as randomized controlled trials in the U.S. and globally. Development of these methods will require extensive review of the literature and collaborations between nutrition researchers, epidemiologists, and meat scientists.

## Figures and Tables

**Table 1:** Descriptions of commonly used methods to measure dietary intake in the U.S.

Method	Description and Examples	Demographic Information
Food Frequency Questionnaires (FFQs)	Relates dietary intake patterns to disease development over time and are designed to yield approximate intakes of broad food categories. Used commonly in prospective cohort studies.	
	An example includes the Harvard T. H. Chan School of Public Health and the Brigham and Women’s Semi-quantitative Food Frequency Questionnaire [10] used in the Health Professional Follow-up Study and Nurses’ Health Study I and II cohorts [17].	At cohort initiation, the Health Professionals Follow-Up Study included *n* = 37,083 men aged 40 to 75 years, the Nurses’ Health Study I included *n* = 79,570 women aged 30 to 55 years, and the Nurses’ Health Study II included *n* = 87,504 women aged 25 to 42 years all with at least 20 years of follow-up data [17].
	Another example includes the National Cancer Institute Diet History Questionnaire II [11] used in the National Institute of Health American Association of Retired Persons (NIH-AARP) Diet and Health Study cohort [3].	At cohort initiation, the NIH-AARP Diet and Health Study included *n* = 536,969 men and women respondents aged 50 to 71 years from six U.S. states and two metropolitan areas with at least 16 years of follow-up data [3].
Food Disappearance Data	Estimates the amount of food in the food supply chain from production to retail outlets available for purchase; used to infer consumption.	
	An example includes the United States Department of Agriculture, Economic Research Service (USDA-ERS) [9] which calculates summary estimates of per capita food and nutrient availability at the primary, retail and consumer levels based on total U.S. population ^1^.	Food availability estimates are developed by utilizing U.S. commodity market information to estimate the national food supply available to the population. The USDA-ERS uses sampling and statistical methods to calculate estimates.
Dietary Recalls	Measures participants’ recollection of food/beverage types and amounts consumed during the previous day; used to infer eating patterns.	
	An example includes the 2013–2014 National Health and Nutrition Examination Survey Food Pattern Equivalency Database (NHANES, FPED) [12] which categorizes and quantifies equivalency (ounces per day ^2^) of dietary recall information from the NHANES into intake of broad food categories.	The 2013–2014 NHANES, FPED database included *n* = 9813 men and women in the U.S. aged 2 to 80 years. Dietary data were collected via two 24-h dietary recalls separated by at least 10 days. Interviews were conducted in-person or by phone using an automated multiple pass method and then statistically analyzed.

^1^ Per capita availability reflects supply at the primary, retail and consumer levels. Loss at the consumer level is accounted for through adjustment and assumptions; more information about adjusting for loss is available at [9] ^2^ In FFQ, 1 ounce ≈ 28 g.

**Table 2:** Comparison of titles and foods included in muscle food categories from the United States Department of Agriculture Economic Research Service (USDA-ERS) and the National Health and Nutrition Examination Survey Food Pattern Equivalency Database (NHANES, FPED) datasets.

NHANES, FPED Dataset ^1^	USDA-ERS Dataset ^2^
**Total meat**	**Total meat, fish, eggs and nuts**
Meat Cured Meat Poultry Organ meat Seafood from high omega-3 sources Seafood from low omega-3 sources	Total red meat Total Poultry Total fresh and frozen, canned, and cured fish and shellfish Eggs Peanuts Total tree nuts Coconut
**Meat**	**Total red meat**
Beef Veal Pork Lamb Game meat	Beef Veal Pork Lamb
**Cured Meat**	**Category not included in dataset**
Cured or luncheon meat made from beef, pork or poultry	
**Poultry**	**Total poultry**
Chicken Turkey Cornish hens Game birds	Chicken Turkey
* Two categories of Seafood	**Total fresh and frozen, canned, and cured fish and Shellfish**
Seafood from high omega-3 sources Finfish Shellfish Other seafood Seafood from low omega-3 sources Finfish Shellfish Other seafood	Total fresh and frozen fish and shellfish Fresh and frozen fish Fresh and frozen shellfish Total canned fish and shellfish Canned salmon Canned sardines Canned tuna Canned shellfish Other canned fish Cured Fish
**Other Categories in the dataset**	**Other Categories in the dataset**
Organ Meat	Eggs
Organ meat from beef, veal, pork, lamb, game or poultry	Peanuts
	Total Tree nuts Almonds Hazelnuts Pecans Walnuts Macadamia nuts Pistachio nuts Other tree nuts
	Coconut

^1^ Adapted from United States Department of Agriculture, Agricultural Research Service, National Health and Nutrition Examination Survey, What We Eat in America, Food Pattern Equivalency Database 2013–2014 dataset available at [12]. ^2^ Adapted from United States Department of Agriculture, Economic Research Service, Food Availability (Per Capita) Data System, Loss-Adjusted Food Availability, Meat, poultry, fish, eggs, and nuts, and fish available at [9]. * Two separate categories of seafood included in dataset; no total seafood category included.

**Table 3:** Questions about muscle foods in the Harvard T.H. Chan School of Public Health and the Brigham and Women’s semi-quantitative food frequency questionnaire ^1^.

Used to Infer Consumption ^2^ by Asking Respondents the Following Prompted Question: “Please Fill in Your Average Total Use, during the Past Year, of Each Specified Food.”
Bacon (2 slices)
Chicken or turkey sandwich or frozen dinner
Other chicken or turkey, with skin (3 ounces)
Other chicken or turkey, including ground without skin (3 ounces)
Beef or pork hot dogs (1)
Chicken or turkey hot dogs or sausages (1)
Salami, bologna, or other processed meat sandwiches
Other processed meats, e.g., sausage, kielbasa, etc. (2 ounces or 2 small links)
Hamburger, lean or extra lean (1 patty)
Hamburger, regular (1 patty)
Beef, pork, or lamb as a sandwich or mixed dish, e.g., stew, casserole, lasagna, frozen dinner, etc.
Pork as a main dish, e.g., ham or chops (4–6 ounces)
Beef or lamb as a main dish, e.g., steak, roast (4–6 ounces)
Liver: beef, calf or pork (4 ounces)
Liver: chicken or turkey (1 ounces)
Canned tuna fish (3–4 ounces)
Breaded fish cakes, pieces, or fish sticks (1 serving, store bought)
Shrimp, lobster, scallops, clams as a main dish (1 serving)
Dark meat fish e.g., tuna steak, mackerel, salmon, sardines, bluefish, swordfish (3–5 ounces)
Other fish, e.g., cod, haddock, halibut (3–5 ounces)

^1^ Adapted from the Harvard T.H. Chan School of Public Health and the Brigham and Women’s semi-quantitative food frequency questionnaire, 2007 version, available at [10], titled “2007 Booklet FFQ”. ^2^ In FFQ, 1 ounce ≈ 28 g.

**Table 4:** Content and calculated percent daily values ^1^ of nutrients from selected unprocessed and processed muscle foods at three intake levels from the USDA Food Composition Database.

Muscle Food	Energy	Protein	Total Fat	Iron		Zinc		Sodium		Potassium		Niacin		B_12_	
	(kcal)	(g)	(g)	(mg)	%DV	(mg)	%DV	(mg)	%DV	(mg)	%DV	(mg)	%DV	(μg)	%DV
Beef, chuck eye steak ^2^															
3 ounces (85 g)	178	23.75	9.17	2.44	14	8.96	60	64	3	323	9	4.41	22	2.86	48
6 ounces (170 g)	355	47.5	18.34	4.88	27	17.92	119	128	5	646	18	8.82	44	5.73	96
9 ounces (255 g)	533	71.25	27.51	7.32	41	26.88	179	191	8	969	28	13.24	66	8.59	143
Beef, tenderloin steak ^3^															
3 ounces (85 g)	168	26.09	7.07	3.05	17	3.98	27	50	2	332	9	5.3	27	3.88	65
6 ounces (170 g)	337	52.19	14.14	6.1	34	7.96	53	100	4	663	19	10.6	53	7.77	130
9 ounces (255 g)	505	78.28	21.22	9.15	51	11.93	80	150	6	994	28	15.9	80	11.65	194
Deli Roast beef ^4^															
3 ounces (85 g)	98	15.83	3.14	1.74	10	2.72	18	725	30	550	16	4.74	24	1.73	29
6 ounces (170 g)	196	31.65	6.27	3.48	19	5.44	36	1450	60	1100	31	9.49	47	3.47	58
9 ounces (255 g)	293	47.48	9.41	5.23	29	8.16	54	2175	91	1650	47	14.23	71	5.2	87
Pork, loin chop ^5^															
3 ounces (85 g)	144	25.96	3.69	0.77	4	2.01	13	57	2	229	7	8.72	44	0.56	9
6 ounces (170 g)	289	51.92	7.38	1.55	9	4.03	27	114	5	457	13	17.45	87	1.12	19
9 ounces (255 g)	433	77.88	11.07	2.32	13	6.04	40	171	7	686	20	26.17	131	1.68	28
Ham, cured ^6^															
3 ounces (85 g)	133	21.29	4.67	0.8	4	2.18	15	1128	47	269	8	4.27	21	0.59	10
6 ounces (170 g)	267	42.59	9.35	1.6	9	4.37	29.	2256	94	537	15	8.53	43	1.19	20
9 ounces (255 g)	400	63.88	14.02	2.4	13	6.55	44	3384	141	806	23	12.8	64	1.78	30
Turkey, breast ^7^															
3 ounces (85 g)	116	25.08	1.67	0.82	5	1.29	9	97	4	252	7	9.99	50	1.5	25
6 ounces (170 g)	231	50.17	3.35	1.65	9	2.58	17	194	8	505	14	19.98	100	2.99	50
9 ounces (255 g)	347	75.25	5.02	2.47	14	3.88	26	291	12	757	22	29.96	150	4.49	75
Deli Turkey ^8^															
3 ounces (85 g)	93	18.54	0.71	0.54	3	1.14	8	660	28	180	5	0.09	0	0.08	1
6 ounces (170 g)	185	37.08	1.41	1.08	6	2.27	15	1320	55	361	10	0.19	1	0.15	3
9 ounces (255 g)	278	55.62	2.12	1.62	9	3.41	23	1980	83	541	15	0.28	1	0.23	4
Salmon ^9^															
3 ounces (85 g)	175	18.79	10.5	0.29	2	0.37	3	52	2	326	9	6.84	34	2.38	0
6 ounces (170 g)	350	37.57	21	0.58	3	0.73	5	104	4	653	19	13.68	68	4.76	1
9 ounces (255 g)	525	56.35	31.49	0.87	5	1.1	7	156	7	979	28	20.52	103	7.14	1
Sardines ^10^															
3 ounces (85 g)	177	20.93	9.73	2.48	14	1.11	7	261	11	337	10	4.46	22	7.6	1
6 ounces (170 g)	354	41.85	19.46	4.96	28	2.23	15	522	22	675	19	8.92	45	15.2	3
9 ounces (255 g)	530	62.78	29.2	7.45	41	3.34	22	783	33	1012	29	13.38	67	22.8	4

^1^ %DV = percent daily value; Calculated by the following: USDA Food Composition Database nutrient amount/U.S. Food and Drug Administration Daily Value Reference * 100. Data adapted from sources [18,19] in Reference list. ^2^ USDA-ARS Food Composition Database number 23077: Beef, chuck eye steak, boneless, separable lean only, trimmed to 0’ fat, all grades, cooked, grilled. ^3^ USDA-ARS Food Composition Database number 13442: Beef, loin, tenderloin steak, boneless, separable lean only, trimmed to 0’ fat, all grades, cooked, grilled. ^4^ USDA-ARS Food Composition Database number 07043: Roast beef, deli style, prepackaged, sliced. ^5^ USDA-ARS Food Composition Database number 10067, Pork, fresh, loin, top loin (chops), boneless, separable lean only, cooked, braised. ^6^ USDA-ARS Food Composition Database number 10153: Pork, cured, ham, whole, separable lean only, roasted. ^7^ USDA-ARS Food Composition Database number 05711, Turkey, retail parts, breast, meat only, cooked, roasted. ^8^ USDA-ARS Food Composition Database number 07046: Turkey breast, low salt, prepackaged or deli, luncheon meat. ^9^ USDA-ARS Food Composition Database number 15237: Fish, salmon, Atlantic, farmed, cooked, dry heat. ^10^ USDA-ARS Food Composition Database number 15237: Fish, sardine, Atlantic, canned in oil, drained solids with bone.

**Table 5:** Examples of red meat categorization in meta-analyses of prospective cohort studies assessing cardiovascular disease risk of consuming 100 g of unprocessed red meat per day.

Author, Year	Categorization and Definitions of Red Meat
Kaluza, 2012 [42]	“fresh red meat”—no definition provided
	“processed meat”—no definition provided
	“total red meat”—no definition provided
Micha, 2010 [39]	“red meat”—unprocessed meat from beef, hamburgers, lamb pork, or game, excluding poultry, fish, or eggs
	“processed meat”—any meat preserved by smoking, curing, or salting or addition of chemical preservatives, including examples such as bacon, salami, sausages, hot dogs, or processed deli or luncheon meats, excluding fish or eggs
	“total meat”—total of these two categories
Micha, 2012 [38]	“total unprocessed red meat”—beef, pork, and lamb
	“total processed meat”—bacon, hot dogs, sausage, salami, and processed deli or luncheon meats
Wang, 2016 [40]	“unprocessed red meat”—beef, lamb, or pork, excluded poultry and fish
	“processed meat”—any meat preserved by salting, curing or smoking, or with the addition of chemical preservatives, including examples such as bacon, sausages, salami, hot dogs or processed deli meats
	“total red meat”—sum of the two categories
Pan, 2012 [37]	“unprocessed red meat”—beef, pork or lamb as main dish, hamburger, and beef, pork, or lamb as sandwich or mixed dish
	“processed red meat”—bacon, hot dogs, sausage, salami, bologna, and other processed red meats

**Table 6:** Categorization of meat in the Dietary Guidelines for Americans (DGA) editions.

DGA Edition and Release Year	Categorization of Meat in the DGA
1st edition; 1980 [44]	Meats, poultry, fish, and eggs
2nd edition; 1985 [45]	Meats, poultry, fish, eggs, and dry beans and peas
3rd edition; 1990 [46]	Meat, poultry, fish, dry beans, and eggs
4th edition; 1995 [47]	Meat, poultry, fish, dry beans, eggs, and nuts group (included in the Food Guide Pyramid)
5th edition; 2000 [48]	Meat, poultry, fish, dry beans, eggs, and nuts group; also referred to as the Meat and Beans (included in the Food Guide Pyramid)
6th edition; 2005 [49]	Meat and Beans (inclusive of poultry and fish as part of eating pattern examples)
7th edition; 2010 [50]	Meat (with separate food groups for poultry and fish as part of eating pattern examples)
8th edition; 2015 [2]	Meats, poultry, and eggs (as part of recommended eating patterns)
